# Diagnosis and treatment of primary central nervous system lymphoma with the primary lesion in the hypothalamus: a case report

**DOI:** 10.1186/s12902-020-00675-5

**Published:** 2021-01-11

**Authors:** Ken Takao, Ayaka Tani, Tetsuya Suwa, Yayoi Kuwabara-Ohmura, Kenta Nonomura, Yanyan Liu, Takehiro Kato, Masami Mizuno, Takuo Hirota, Mayumi Enya, Katsumi Iizuka, Yukio Horikawa, Chiemi Saigo, Yusuke Kito, Tatsuhiko Miyazaki, Naoyuki Ohe, Toru Iwama, Daisuke Yabe

**Affiliations:** 1 Department of Diabetes and Endocrinology, Gifu University Graduate School of Medicine, 1-1 Yanagido, Gifu, 501-1194 Japan; 2 Division of Pathology, Gifu University Hospital, Gifu, Japan; 3 Department of Neurosurgery, Gifu University Graduate School of Medicine, Gifu, Japan

**Keywords:** PCNSL, Hypothalamus, Hypopituitarism, Case report

## Abstract

**Background:**

Primary central nervous system lymphoma is a rare extra-nodal lymphoma of the central nervous system. Primary central nervous system lymphoma lesions usually appear in the vicinity of the ventricle, and there are few reports of primary central nervous system lymphoma with hypothalamic-pituitary lesions.

**Case presentation:**

We treated a 56-year-old male with primary central nervous system lymphoma with the primary lesion in the hypothalamus, which was found by magnetic resonance imaging after sudden onset of endocrinological abnormalities. Initially, he was hospitalized to our department for hyponatremia. Endocrinological examination in conjunction with head magnetic resonance imaging and endoscopic biopsy revealed hypothalamic hypopituitarism and tertiary hypoadrenocorticism caused by a rapidly growing, diffuse large B-cell lymphoma in the hypothalamus. Remission of the tumor was achieved by high-dose methotrexate with whole brain radiotherapy, and some of the hormone responses were normalized.

**Conclusions:**

While primary central nervous system lymphoma is rare, it is important to note that hypopituitarism can result and that the endocrinological abnormalities can be partially restored by its remission.

## Background

Primary central nervous system lymphoma (PCNSL) is an extra-nodal lymphoma that shows no lesions other than in the central nervous system at the time of diagnosis [1]. While the disease is relatively rare, comprising 1–5% of brain tumors, the number of PCNSL cases is increasing recently [2, 3]. PCNSL is frequently found in association with acquired immunodeficiency syndrome (AIDS) in western countries; most PCNSL cases in Japan are without AIDS and tend to be found in individuals age 50–70 in whom the disease progresses from B lymphocytes [4]. PCNSL lesions usually appear in the vicinity of the ventricle, and are often multiple. Initial symptoms may include subclinical intracranial pressure and psychiatric conditions in addition to other conditions originating in the brain such as paralysis and aphasia [5]. PCNSL with hypothalamic-pituitary lesions has been rarely reported [6]. Here we report a case of PCNSL with the primary lesion in the hypothalamus, which was found by magnetic resonance imaging (MRI) after sudden onset of endocrinological abnormalities.

## Case presentation

At 56 years of age, the patient was diagnosed with Connshing syndrome and received laparoscopic left adrenalectomy at another medical institution. He then started receiving hydrocortisone (HC) replacement and was monitored for ACTH and cortisol levels. He was also diagnosed with an old myocardial infarction and started receiving aspirin (100 mg/day). At 57 year of age, he was transferred to our hospital due to his residential relocation. When he visited our hospital for the first time, his ACTH and cortisol ≥24 h after taking 2.5 mg HC the previous morning were 48.6 pg/ml and 15.2 μg/dl, respectively, and his HC replacement was terminated.

**Fig. 1:**
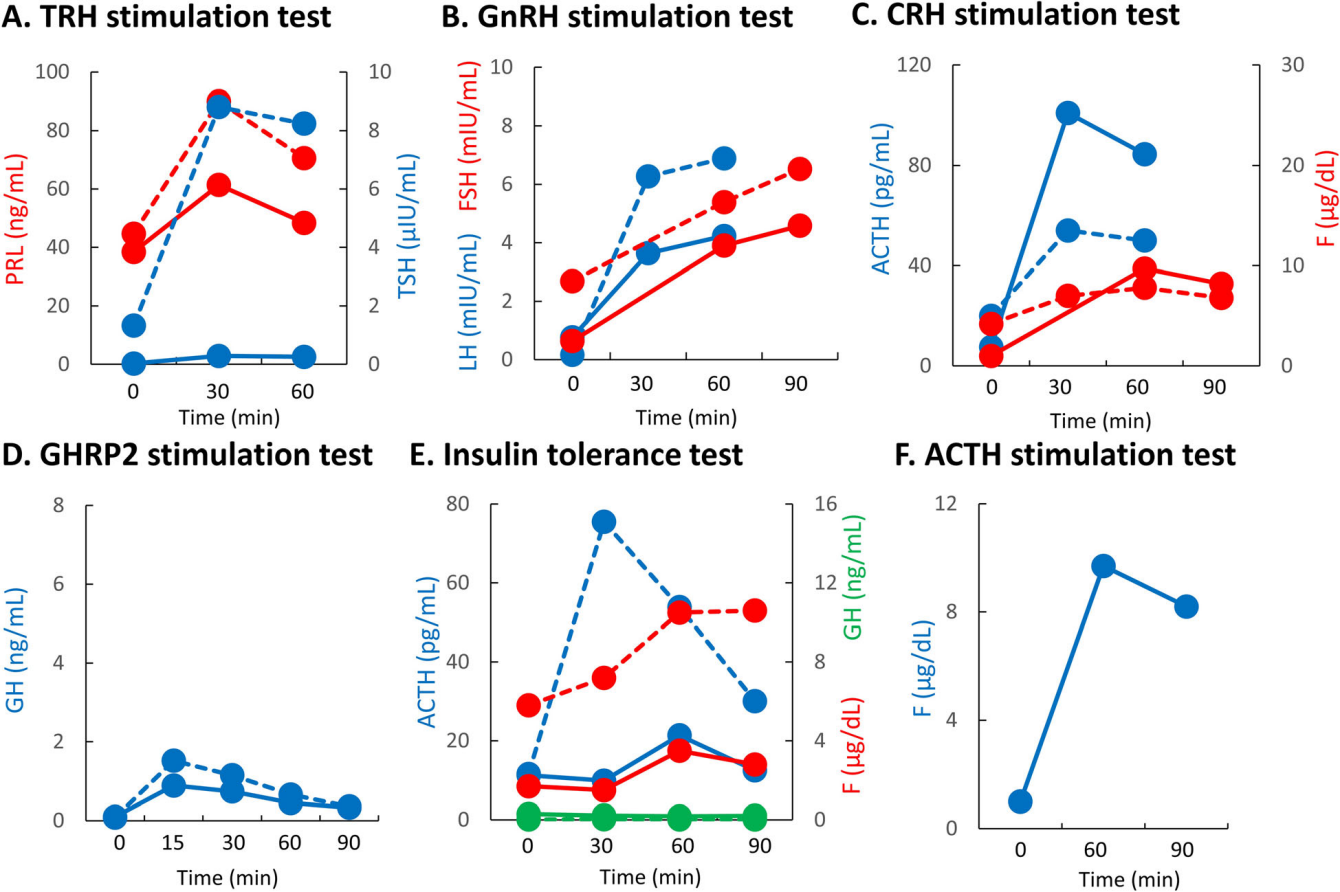
Results of endocrinological examinations. Various endocrinological stimulation tests suggest the presence of hypothalamic hypopituitarism and tertiary hypoadrenocorticism in the patient. **a**. TRH stimulation test. **b**. GnRH stimulation test. **c**. CRH stimulation test. **d**. GHRP2 stimulation test. **e**. Insulin tolerance test. **f**. ACTH stimulation test. Solid lines represent changes in hormone levels before the treatment of primary central nervous system lymphoma; dashed lines represent those after the treatment

**Fig. 2:**
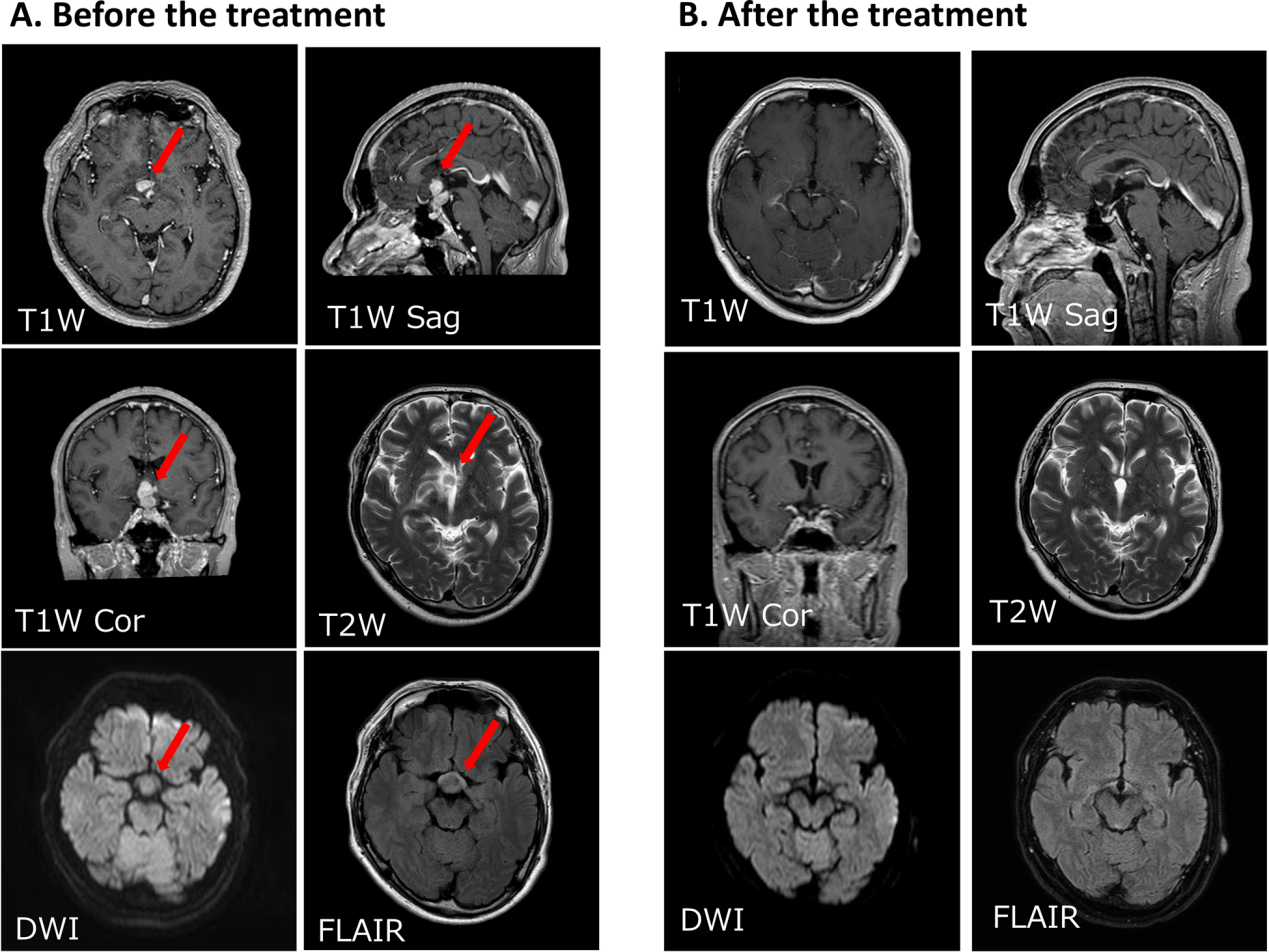
Imaging analysis before and after the treatment of the central nervous system lymphoma. **a**, **b**. Gadolinium-enhanced magnetic resonance imaging (MRI) before and after the initial treatment of the patient’s central nervous system lymphoma. A 16 × 12 mm lobulated tumor mass in the hypothalamus that extends to the pituitary stalk and the right temporal lobe was detected as a low intensity mass in T2-weighted imaging, a faint high intensity mass in diffusion-weighted imaging, and fluid attenuated inversion recovery imaging (**a**). The mass was no longer detected after the treatment (**b**). T1, T1-weighted imaging; T2, T2-weighted imaging; DWI, diffusion-weighted imaging; FLAIR, fluid attenuated inversion recovery imaging; Sag, sagittal plane; Cor, coronal plane

**Fig. 3:**
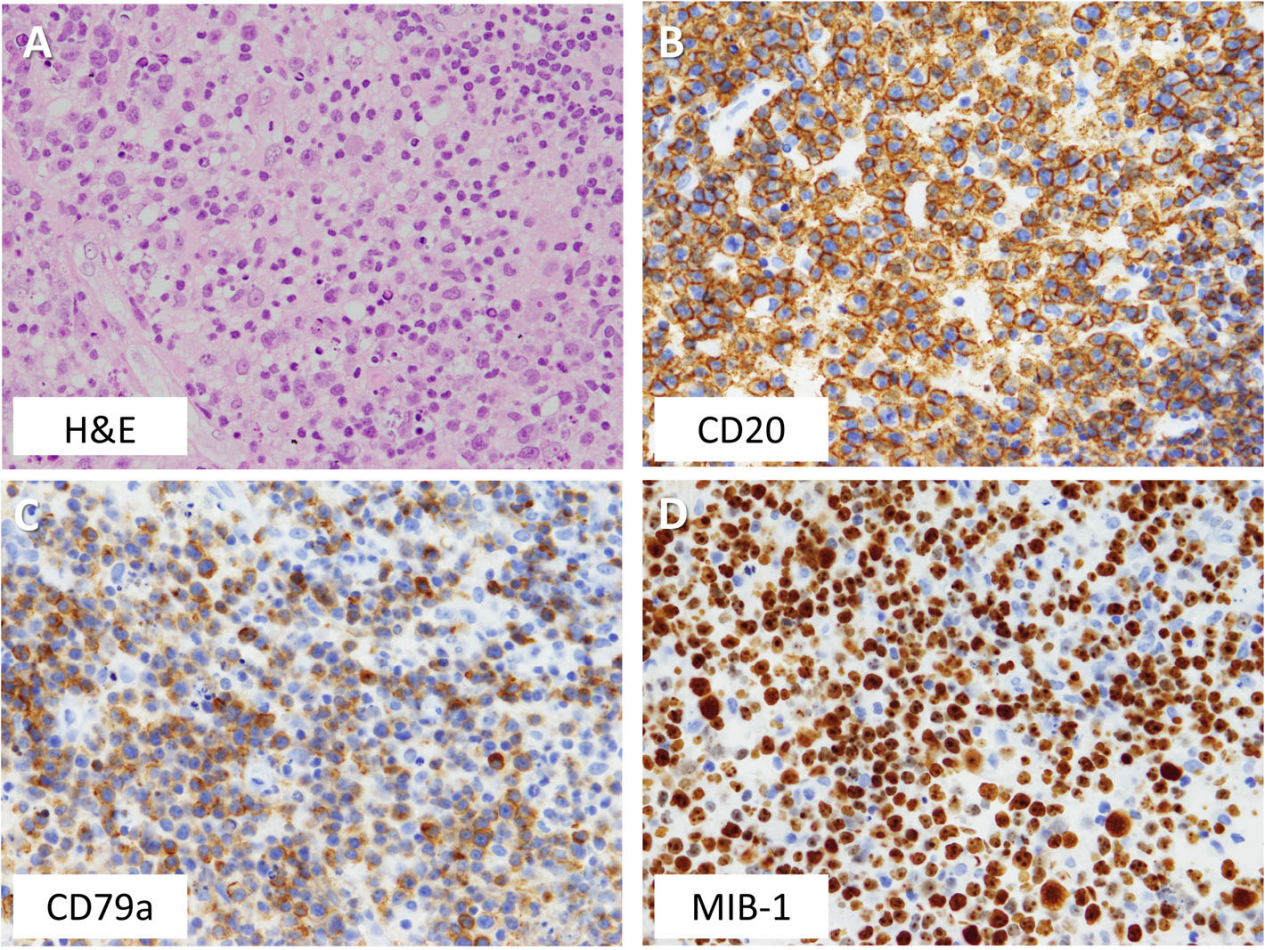
Pathological analysis of the patient’s tumor. The analysis found proliferation of atypical lymphocytes that were positive for CD20 and CD79a. MIB-1 index was approximately 80%. Magnification × 400

Three months later, he began to suffer from blurred vision, headache and appetite loss, and was admitted to the emergency department of our institution. His height and body weight were 164.8 cm and 76.3 kg, respectively. His vital signs were normal (blood pressure, 129/93 mmHg; pulse 82 bpm; body temperature 36.7 °C; SpO_2_ 96%) and he showed no abnormalities in neurological or physical examinations that we routinely perform in clinical practice. However, his serum sodium concentration was 106 mEq/L and plasma osmolality was 217 mOsm/kgH_2_O (Tables 1 and 2), and he was then hospitalized to our department. After hospitalization, his hyponatremia was corrected by intravenous and oral administration of sodium chloride, which, together with HC replacement from the 3rd day after hospitalization, improved his blurred vision, headache and appetite loss. In the morning of the 2nd day after hospitalization, levels of TSH, ACTH, GH, LH and FSH were low, and that of PRL was high (Table 3). On the 2nd day after hospitalization, his urinary cortisol was ≤15.5 μg/dL, while plasma levels of ACTH (pg/mL) and cortisol (μg/dL) were as follows: 8 am, 17.5 and 1.4; 2 pm, 13.7 and 1.0; 8 pm 14.1 and 1.1; and 11 pm 8.8 and 0.8, respectively. Little TSH and GH response was observed in TRH and GHRP-2 stimulation tests, respectively (Fig. 1a and d). Near normal LH and FSH responses were observed in GnRH stimulation test (Fig. 1b), but basal levels of LH and FSH were low (Table 3). Hyper ACTH response with low cortisol response was observed in CRH stimulation test (Fig. 1c). ACTH response was delayed with a low cortisol response and little GH response in insulin stimulation test (Fig. 1e). Peak cortisol level was < 18 μg/dL in ACTH rapid stimulation test, which was carried out ≥24 h after administration of 10 mg HC (Fig. 1f). These results indicated the presence of hypothalamic hypopituitarism and tertiary hypoadrenocorticism. The patient then experienced atypical visual field loss in his right eye. His MRI on day 14 after hospitalization revealed a rapidly growing tumor in the hypothalamus (Fig. 2a) that was absent 1 year before. Endoscopic biopsy of the tumor using a ventriculoscope revealed a proliferation of atypical lymphocytes, which was positive for CD20 and CD79a (Fig. 3) as well as CD10 and Bcl-6 but negative for Bcl-2 (data not shown). The MIB-1 index was approximately 80% (Fig. 3). These results together suggested B-cell lymphoma. His serum level of soluble IL-2 receptor was 436.0 U/mL (normal range, 122–496 U/mL), and examination of cerebrospinal fluid revealed no abnormalities.

**Table 1:** Biochemistry and complete blood count upon admission to our emergency department

Biochemistry				Complete blood count	
Total protein	7.6 g/dL	Cre	0.48 mg/dL	WBC	5830 /μL
Albumin	4.5 g/dL	BUN	5.3 mg/dL	Neutro	51.9%
CPK	358 U/L	Total-cho	171 mg/dL	Mono	5.0%
AST	46 U/L	HDL-cho	30 mg/dL	Lymph	34.0%
ALT	77 U/L	TG	205 mg/dL	Eosino	8.6%
LDH	211 U/L	Na	106 mmol/L	Baso	0.5%
ALP	415 U/L	K	4.2 mmol/L	RBC	486× 10^4^ / μL
γ-GTP	115 U/L	Cl	77 mmol/L	Hb	14.6 g/dL
CHE	275 U/L	Ca	9.2 mg/dL	Plt	29.5×104 /μL
AMY	42 U/L	IP	2.4 mg/dL		
UA	2.3 mg/dL	BNP	42.9 pg/mL		
CRP	0.74 mg/dL	Glu	127 mg/dL

**Table 2:** Urinalysis upon admission to our emergency department

Specific gravity	1.011
pH	6.0
Protein	–
Glucose	–
Ketone body	+
Blood	+
Osmolarity	439 mOsm/kgH_2_O
Na	148 mEq/L
K	36.6 mEq/L
Cl	128 mEq/L

**Table 3:** Basal levels of various hormones after normalization of hyponatremia

TSH	0.06 μIU/mL	PRL	28.9 ng/mL
free T3	2.8 pg/mL	LH	≤0.10 mIU/mL
free T4	0.71 ng/dL	FSH	0.44 mIU/mL
ACTH	4.2 pg/mL	PRA	0.9 ng/mL/hr
Cortisol	7.9 μg/dL	PAC	233.0 pg/mL
GH	0.05 ng/mL		
IGF-1	88 ng/mL (−1.8 SD)

He then started 6 courses of high-dose methotrexate (3.5 g/m^2^) with stereotactic radiotherapy without whole brain radiotherapy (20 Gy) along with oral administration of HC (15 mg/day) and levothyroxine (25 μg/day), which resulted in remission of the tumor (Fig. 2b). Some of the hormone responses (i.e., ACTH, TSH, PRL, LH and FSH), which were determined were normalized (Fig. 1).

**Fig. 4:**
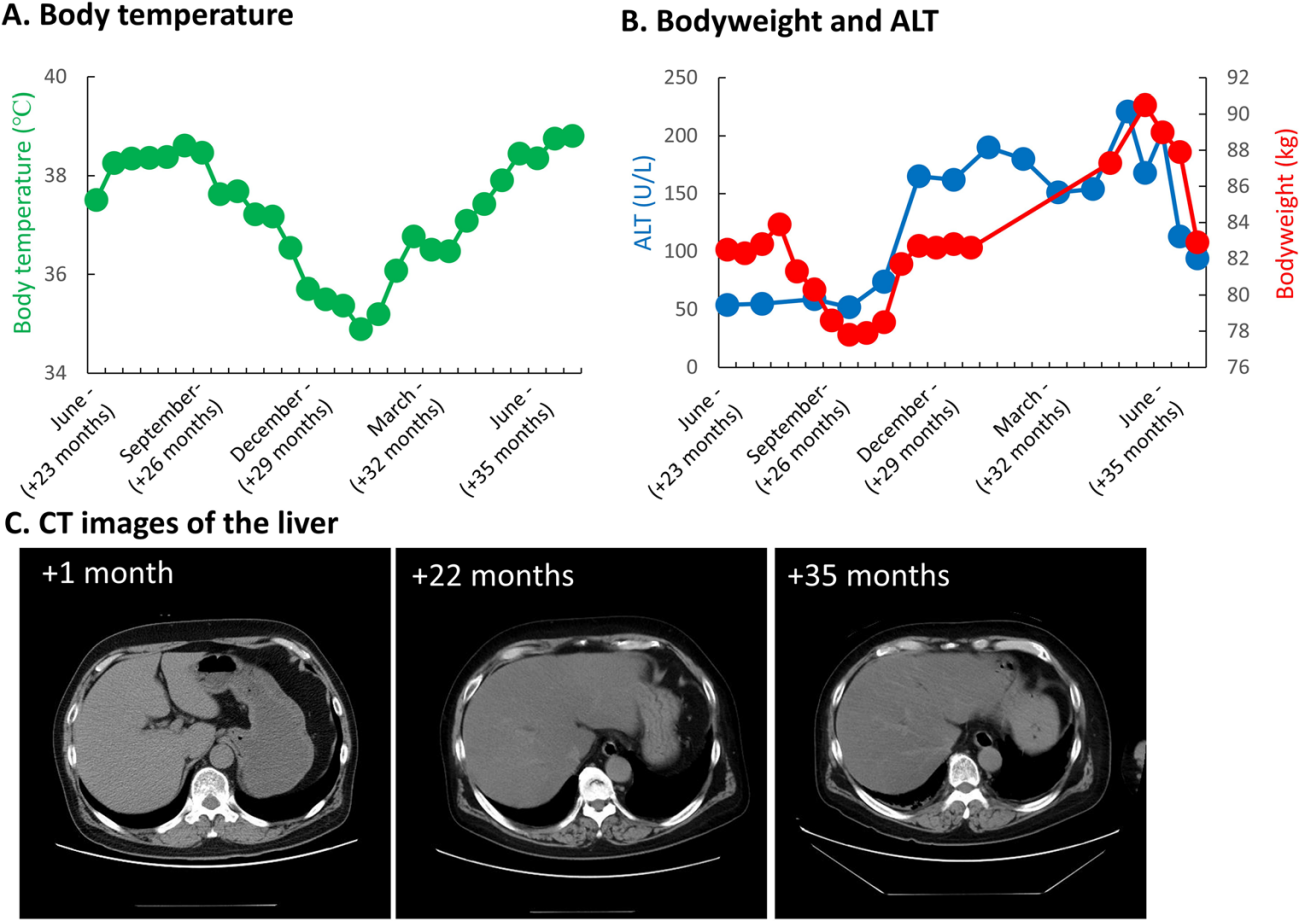
Changes of body temperature, body weight and serum levels of alanine aminotransferase (ALT) and computer tomography (CT) images of the patient’s liver. **a**. His body temperature changed with the rise and fall of the ambient temperature, likely due to the impaired thermoregulatory center. **b**. His body weight and ALT level gradually became elevated, likely due to dysregulated appetite. **c**. The ratio of his liver-to-spleen (L/S) Hounsfield units on CT scan gradually declined (+ 1 month, 0.96; + 22 months, 0.42; and + 35 months, 0.37), likely due to worsening fatty liver [months after the initial hospitalization to our institution are shown as +X month(s)]

Eight months after the initial hospitalization, he had a relapse of the tumor and received 3 courses of high dose methotrexate with whole brain radiotherapy (23.4 Gy), which resulted in another remission of the tumor. Twenty-two months after the initial hospitalization, he was hospitalized due to profound hyperthermia and disturbance of consciousness. Investigations including head MRI failed to find a cause of the hyperthermia, and hypothalamic dysfunction was suspected. His body temperature was high during summer and low during winter (Fig. 4a). He had increased appetite likely due to hypothalamic dysfunction, which resulted in body weight gain and fatty liver (Fig. 4b and c). He then started showing a gradual decline in cognitive function and activities of daily living. His head MRI at regular checkup revealed recurrence of the PCNSL at 38 months after the initial hospitalization; the patient died 39 months after the initial hospitalization.

## Discussion and conclusions

Here we present a case of PCNSL with the primary lesion in the hypothalamus, which was found by MRI and assumed to be the cause of his hypopituitarism. While the patient’s age and pathological findings are consistent with typical PCNSL cases, this case is exemplified by hyponatremia due to hypoadrenocorticism, which together prompted the diagnosis and reflect the localization of the tumor. PCNSL with hypothalamic-pituitary lesions is extremely rare [7]. Symptoms of hypopituitarism may appear suddenly and quickly worsen as the tumor grows rapidly, as in the current case [8]. Indeed, we found a rapid decline in adrenal cortical function over a relatively short period of about 3 months. Importantly, secretions of some pituitary hormones (e.g., ACTH, TSH, PRL, LH and FSH) during various stimulation tests were partially improved by remission of the tumor, further suggesting infiltration and displacement of the hypothalamus and pituitary stalk as the cause of hypopituitarism in this case.

Risk factors for PCNSL include chronic use of immunosuppressants for organ transplants and autoimmune diseases in addition to immunodeficiency associated with HIV infection [9, 10]. While the current case had been on HC replacement therapy before admission to our institution, because of the short duration and low dosage it is not likely that he was immunosuppressed. We did not investigate genetic alterations in the current case, but previous studies have shown the B-cell receptor pathway, which involves MYD88, CD79B and NF-kB activation, to be critical in the pathogenesis of PCNSL [11].

In conclusion, we treated a case of PCNSL with the primary lesion in the hypothalamus, which was found by MRI after sudden onset of endocrinological abnormalities. While PCNSL is rare, it is important to note that hypopituitarism can be caused by the disease and that endocrinological abnormalities can be partially restored by its remission.

## Data Availability

Clinical data from the corresponding author will be available upon request.

## Abbreviations

AIDS: Acquired immunodeficiency syndrome
MRI: Magnetic resonance imaging
PCNSL: Primary central nervous system lymphoma
HC: Hydrocortisone
